# Triboelectric Nanogenerator-Based Self-Powered Resonant Sensor for Non-Destructive Defect Detection

**DOI:** 10.3390/s19153262

**Published:** 2019-07-24

**Authors:** Jinkai Chen, Chi Zhang, Weipeng Xuan, Liyang Yu, Shurong Dong, Yuedong Xie, Wuliang Yin, Jikui Luo

**Affiliations:** 1 Ministry of Education Key Lab. of RF Circuits and Systems, College of Electronics & Information Hangzhou Dianzi University, Hangzhou 310000, China; 2 Key Lab of Advanced Micro/Nano Electronic Devices & Smart Systems of Zhejiang, College of Information Science & Electronic Engineering, Zhejiang University, Hangzhou 310000, China; 3 School of Electrical and Electronic Engineering, University of Manchester, Manchester M60 1QD, UK; 4 Institute of Renewable Energy & Environmental Technology, University of Bolton, Deane Road, Bolton BL3 5AB, UK

**Keywords:** triboelectric, self-powered sensor, non-destructive testing, resonant

## Abstract

A triboelectric nanogenerator-based self-powered resonant sensor is proposed and investigated. By integrating an inductor and a microswitch with a triboelectric nanogenerator, a new type triboelectric nanogenerator is obtained, the pulse voltage output is converted to an oscillating signal with a very stable modulated resonant frequency, immune to the cross disturbance of contact-related variation (force, frequency, distance) and environmental variation, such as humidity and temperature. This is utilized for non-destructive defect detection. When the coil inductor scans the surface of a specimen with defects, varying resonant frequencies are obtained for different types of defects, showing excellent consistency between the experimental and simulated results. The results demonstrate the potential of the self-powered TENG-based resonant sensor to be a highly stable and sensitive magnetic sensor for the non-destructive defect detection applications.

## 1. Introduction

Nowadays, the Internet of things (IoT), which consists of trillions of sensors [1], has attracted ever increasing attention as an emerging technology. It is unrealistic to power such huge numbers of sensors using batteries due to periodic maintenance and replacement issues. Therefore, developing an energy solution suitable for IoT becomes one of the most urgent tasks. Energy harvesting from the ambient environment using nanogenerators have emerged as one of the best ways to power the sensors, and it enables the sensor to be maintenance-free. Piezoelectric [2,3,4], pyroelectric [5,6,7], and triboelectric [8,9,10] effects based nanogenerators have been explored as power sources or self-powered sensors. Among them, the triboelectric nanogenerator (TENG) has been regarded as one of the most promising technologies owing to its high energy output and conversion efficiency, simple device structure and fabrication process, and low cost.

TENG utilizes contact and separation of two materials with dissimilar electron affinities to convert mechanical energy, such as human motion [11,12,13], rain dropping [14,15,16], or wind blowing [17,18,19], into electric energy. The output voltage of TENGs is highly sensitive to motion-related variables [20,21] including contact force, frequency and distance between two tribo-materials and environment-related variables [22,23,24], such as humidity, temperature, and pressure. As such, TENGs have been utilized not only as energy harvesting devices but also as self-powered sensors for various applications. One of the main shortcomings of TENG-based self-powered sensors is the cross disturbance or influence of different variables, which makes the performance of the sensors inaccurate and unstable. The environmental variation (humidity, temperature, or pressure, etc.) or contact variation (force, frequency, or spacer distance, etc.) cross disturbance can be minimized by using complicated package process [25] or highly specified contact setup [26] to improve the sensing accuracy, respectively. Although the cross-influence can be minimized to some extent using the above-mentioned techniques, new and innovative methods are yet to be developed. Here we propose a triboelectric nanogenerator-based self-powered resonant sensor, which integrates an inductor with a microswitch enhanced TENG device. Based on our previous work [26], a very stable TENG resonant voltage output is obtained, which is immune to the cross disturbance of contacting and environmental variation, such as humidity. Such a stable voltage signal from the self-powered resonant sensor is then utilized for non-destructive defect testing (NDT) with excellent agreement with modeling results, demonstrating its great potential for applications.

## 2. Results and Discussion

### 2.1. Materials and Methods

**Figure 1:**
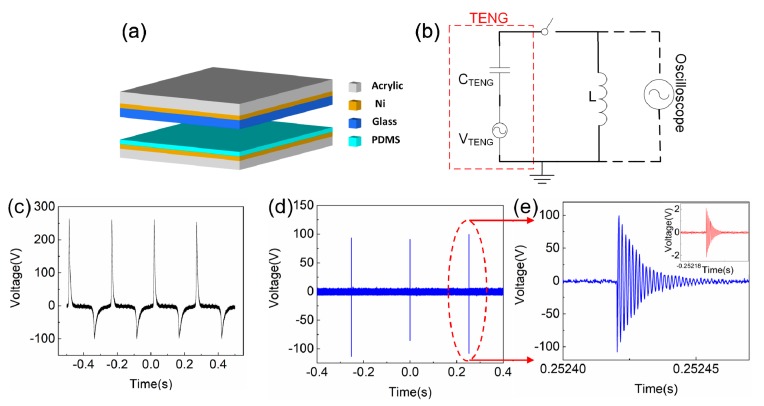
TENG structure (**a**), the equivalent circuit for the sensor system with a microswitch (**b**), open-circuit (~100 MΩ external load) voltage output (**c**), the boosted and stabilized voltage output (**d**), and the zoom-in resonant signal details with and without (inset) the microswitch (**e**).

**Figure 2:**
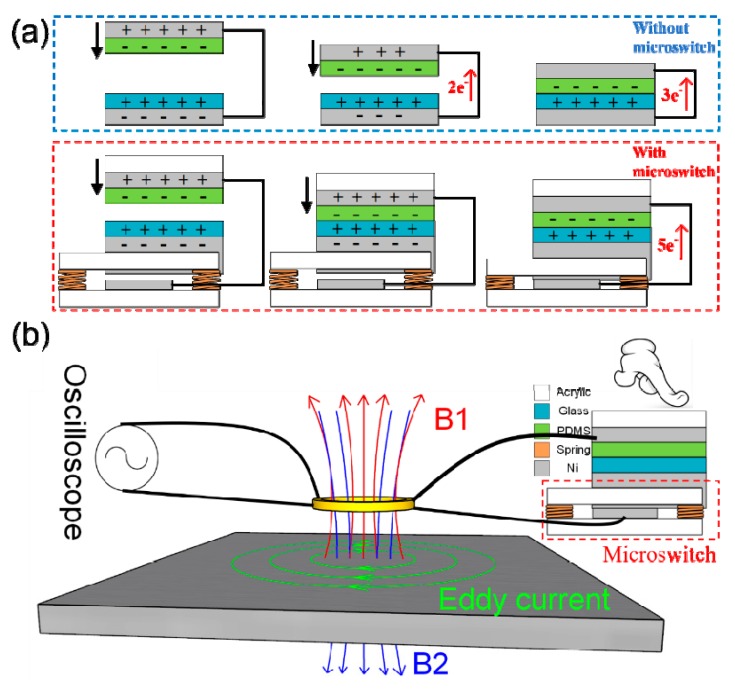
(**a**) Charge transfer process with and without the enhancement of microswitch and (**b**) schematic of the proposed TENG-based resonant sensor for non-destructive defect detection.

The structure of the TENG used in this study is similar to that shown in our previous work [26], as shown in Figure 1a. It consists of a positive tribo-material of glass and a negative tribo-material of polydimethylsiloxane (PDMS) with a dimension of 4.5 × 4.5 cm^2^. The PDMS (184 Silicone Elastomer, Dow Corning Co. Ltd., CA, USA) solution is a mixture of base PDMS and cure agent (10:1 by mass). The solution was spin-coated on a double-side Ni adhesive tape at 1100 rpm for 10 s, which results in a PDMS film thickness of ~100 μm. The Ni tape with PDMS film was attached on an acrylic support layer and then cross-linked at 100 °C on a hot plate for 25 min. The glass part of TENG contains a commercial 1 mm soda lime glass, which was attached to an acrylic support layer through Ni adhesive tape. A simplified illustration of TENG with a microswitch is shown in Figure 2b, and the PDMS part is as it is in Figure 1a, while for the glass part, the acrylic support layer was replaced with a microswitch, which consists of four springs and two Ni conductive layers attached to two acrylic support layers separately, and the distance between two Ni conductive layer is ~400 μm. A linear motor was utilized to control the cyclic contact of TENG, and the output voltage of TENG was obtained using an oscilloscope (Keysight MSO9254A).

Figure 1c illustrates the typical “open-circuit” (~100 MΩ external load) voltage output for the TENG, which was measured under 4 Hz contact frequency, 16 mm spacer, 40 N force, and 60% environment humidity, and the positive and negative peak amplitudes were 261 V and −101 V, respectively. In addition, it can be seen that the signal width of the voltage output is more than 30 ms. When an inductor L is connected in series with the TENG, as shown in Figure 1b, it forms an oscillating circuit, and the pulsed output voltage can be converted into a short period (~50 μs) oscillating signal with the resonant frequency, *f*, determined by the inductor (*L*) and capacitor (*C*)of the circuit as follows:

(1) $$f=\frac{1}{2\pi \sqrt{LC_{TENG}}},$$

where *C_TENG_* is the capacitor of the TENG, which can be expressed as:

(2) $$C_{TENG}=\frac{\varepsilon_{0}S}{d_{0}+x},$$

where *ε*_0_, *S*, and *x* represent the vacuum permittivity, contact area, and distance between two tribo-materials, respectively. *d*_0_ is the effective TENG thickness, which is defined as:

(3) $$d_{0}=\frac{d_{1}}{\varepsilon_{r1}}+\frac{d_{2}}{\varepsilon_{r2}},$$

where *d*_1_ and *d*_2_ are the thickness, *ε_r_*_1_ and *ε_r_*_2_ are the relative permittivity of the PDMS and glass, respectively. However, the amplitude of the oscillating signal on the inductor is extremely small (<10 V) due to the large impedance mismatch between the TENG and inductor [26,27]. We found that the amplitude of the oscillating signal can be increased and the resonant frequency can be stabilized significantly by integrating a synchronization microswitch with the LC circuit, as shown in Figure 1b. The microswitch enhanced oscillating voltage signal measured from the inductor was increased to ~200 V peak to peak amplitude, as shown in Figure 1d, which is ~50 times larger than that without the microswitch (~4 V) as shown in Figure 1e inset. Meanwhile, the signal width is reduced from 30 ms (Figure 1c) to ~50 μs (Figure 1e).

### 2.2. Working Principle of the Microswitch Integrated TENG

The improvement is attributed to the microswitch with the structure shown in Figure 2b, and the charge transfer process with and without the enhancement of microswitch, as shown in a. For the TENG without the microswitch, when the two tribo-materials get closer, the charges (*Q*) transfer gradually (first two electrons then three electrons, as shown the Figure 2a) from the glass side to the PDMS side to maintain the electrostatic equilibrium, leading to a small current/voltage output following the rules of $I=\frac{dQ}{dt}$ and V=IR. When a microswitch is integrated with the TENG, the inductive charges cannot be discharged and will accumulate in the electrodes as two tribo-materials get closer, and the microswitch will only be switched on synchronously after the two tribo-materials contact each other. Although the total generated/transferred charges are the same, it results in an instantaneous discharge of the inductive charges (five electrons, as shown in Figure 2a) and boosted current/voltage outputs [26]. Moreover, for the integrated microswitch case, when the microswitch is on, and the voltage output signal is generated, the TENG’s capacitance is a constant according to Equations (2) and (3) as the distance between the two tribo-materials is zero no matter what the contact variation or how much the humidity is, thus a very stable resonant frequency of the resonant sensor immune from the cross disturbance of contacting and environment is obtained. According to our previous work [26], the fluctuation in resonant frequency can be controlled at a level of less than 1%, which is a significant improvement compared with voltage amplitude outputs based TENGs. Therefore, the self-powered resonant sensor can be utilized in various sensing applications.

### 2.3. Self-Powered Resonant NDT Sensor

Since the resonant frequency of the microswitch enhanced TENG voltage output is no longer influenced by the contact variation and humidity, the oscillating signal of the resonator can be modulated by external passive LC sensors. Here we propose a new type of self-powered resonant sensor for non-destructive defect testing in metals, which is similar to the Eddy current sensor for defect detection in metal [28,29,30], yet it is a self-powered and standalone chipless sensor (it is so called as the sensor system contains no microelectronic devices and chips).

As shown in Figure 2b, the principal for an Eddy current sensor is that alternating current excitation in a coil inductor induces a magnetic field (B1), leading to an eddy current in the test specimen, which establishes an opposed magnetic field (B2), resulting in a net flux linkage of the coil inductor and hence, the impedance (inductance) of coil. Any defects in the test specimen will affect the magnetic field induced by the coil, which alternates the impedance (inductance) of the coil inductor. In the self-powered resonant sensor, the TENG provides the resonant circuit with a pulsed energy, and the resonant frequency of the oscillating signal will change due to the impedance shifting of the coil if there is a defect in the specimen. The sensor requires no external electricity (A.C. excitation signal) to operate. By moving the coil on the surface of the specimen, it is possible to detect the distribution of defects in the specimen.

**Figure 3:**
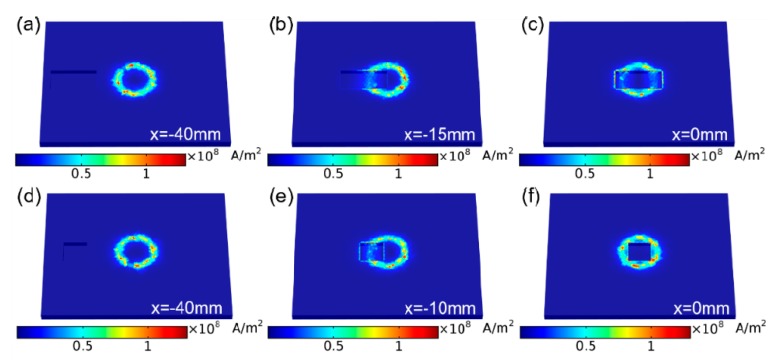
Finite element analysis of the current density distribution on Al plate specimen when the larger defect (30 mm × 15 mm × 3 mm) (**a**–**c**) and smaller defect (15 mm × 15 mm × 3 mm) (**d**–**f**) moving from leftmost position (*x* = −40 mm) to the center (*x* = 0 mm).

Finite element analysis (FEA) was conducted using COMSOL Multiphysics software before experiments to see if this innovative sensor will work or not. The specimen for the modeling was an aluminum (Al) metal plate (100 mm × 120 mm × 5 mm, electrical conductivity = 3.538 × 10^7^ S/m) with two types of defects on its surface. The defects have dimensions of 30 mm × 15 mm × 3 mm and 15 mm × 15 mm × 3 mm, respectively. A Cu-coil (electrical conductivity = 6 × 10^7^ S/m) was used for simulation with 100 turns. The outer radius of the coil was 13 mm, and the inner diameter of the coil was 10.5 mm with a height of 2 mm. The lift-off between the coil and the Al plate was 1 mm. For simplicity of simulation, the defect on the metal plate was moving, and the position of the coil was fixed. The simulated absolute magnitude of current density distributions on the Al plate specimen is illustrated in Figure 3 when the defect moves from the leftmost position (*x* = −40 mm) to the central position (*x* = 0 mm). For the larger defect (30 mm × 15 mm × 3 mm), it can be seen that the strongest current density occurs when the defect is distanced from the coil (*x* = −40 mm, Figure 3a), and it decreases when the defect moves closer to the coil (*x* = −15 mm, Figure 3b). At last, the weakest current density occurs when the defect aligns with the center of the coil (*x* = 0 mm, Figure 3c). The obtained results are different for the small defect (15 mm × 15 mm × 3 mm), which has almost the same current density at the leftmost (*x* = −40 mm, Figure 3d) and center (*x* = 0 mm, Figure 3f), but much weaker current density at *x* = −10 mm position as the defect interacts with the coil physically, resulting in a small eddy current and weaken the sensing current density. The simulated results have clearly demonstrated the proposed self-powered resonant sensor can be used for non-destructive detection.

**Figure 4:**
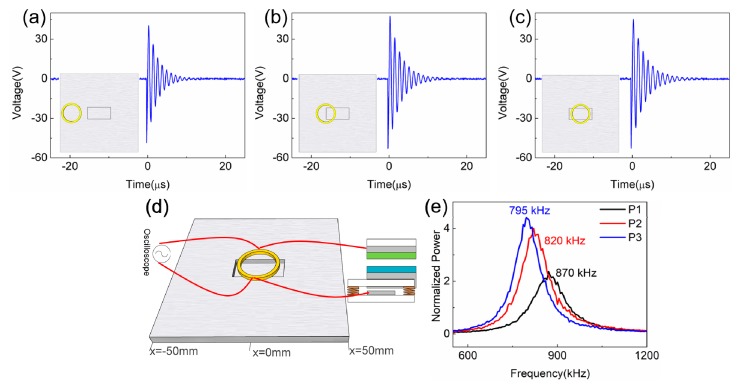
Comparison of resonant signals collected from the coil when it is away from the defect (30 mm × 15 mm × 3 mm) at P1 (**a**), half at P2 (**b**), and on top of the defect at P3 (**c**). (**d**) Experiment configuration of non-destructive defect detection using microswitch integrated TENG and the coil inductor. (**e**) is the comparison of the FFT spectra for three cases, showing the variation of the resonant frequency at P1, P2, and P3.

For the experimental setup, as shown in Figure 4d, the TENG and resonant sensor have the same configuration and setup as those used in the simulation except that the coil is moved to scan the fixed defect on Al plate specimen. A linear motor was utilized to control the cyclic contact for the microswitch integrated TENG. An oscilloscope was connected to the inductor to record the sensing signal. The saved signals can be further analyzed using fast Fourier transformation (FFT) to obtain the spectrum of the signals. Figure 4a–c are the comparison of the resonant signals collected from the coil when the coil is at x = −32.5 mm (P1), x = −12.5 mm (P2), and x = 2.5 mm (P3) position from the 30 mm ×15 mm ×3 mm defect, respectively. It can be noted that the amplitude of the TENG voltage outputs appears very similar. The FFT spectra of the resonant signals are shown in Figure 4e, and the detected signals clearly show the variation of the resonant frequency when the coil is in different positions, which decreases from 870 kHz to 795 kHz when the coil moves from P1 to P3.

For the experimental setup, as shown in Figure 4d, the TENG and resonant sensor have the same configuration and setup as those used in the simulation except that the coil is moved to scan the fixed defect on Al plate specimen. A linear motor was utilized to control the cyclic contact for the microswitch integrated TENG. An oscilloscope was connected to the inductor to record the sensing signal. The saved signals can be further analyzed using fast Fourier transformation (FFT) to obtain the spectrum of the signals. Figure 4a–c are the comparison of the resonant signals collected from the coil when the coil is at x = −32.5 mm (P1), x = −12.5 mm (P2), and x = 2.5 mm (P3) position from the 30 mm ×15 mm ×3 mm defect, respectively. It can be noted that the amplitude of the TENG voltage outputs appears very similar. The FFT spectra of the resonant signals are shown in Figure 4e, and the detected signals clearly show the variation of the resonant frequency when the coil is in different positions, which decreases from 870 kHz to 795 kHz when the coil moves from P1 to P3.

**Figure 5:**
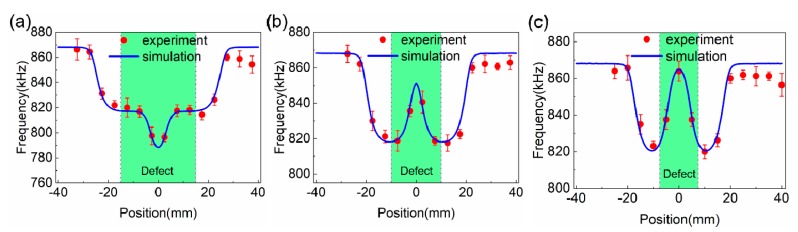
Comparison of experiment and simulation resonant frequency results of non-destructive scanning using a large size defect (30 mm × 15 mm × 3 mm) (**a**), a middle size defect (20 mm × 15 mm × 3 mm) (**b**), and a small size defect (15 mm × 15 mm × 3 mm) (**c**), and the green area indicates where the defects located.

Figure 5a–c are a comparison of the experimental and simulation resonant frequency results of non-destructive scanning over a large size defect (30 mm × 15 mm × 3 mm), a middle size defect (20 mm × 15 mm × 3 mm), and a small size defect (15 mm × 15 mm × 3 mm), respectively. The theoretical resonant frequencies were calculated using Equation (1) with the FEA simulated coil inductance and constant *C_TENG_* [26]. It is clear that the experimental and simulation results fit very well for the non-destructive defect testing, showing a completely different frequency shift trend when scanning the large and small size defects. These are consistent with the discussion for Figure 3. It should be noted that the experimental resonant frequency shows a downward trend once the coil passes the defect (*x* around 30–40 mm), which should be the same as the initial resonant frequency when coil at the leftmost position (*x* = −40 mm) according to the simulation results. It can be attributed to the Al plate size difference between experiment (100 mm × 100 mm × 5 mm) and simulation (100 mm × 120 mm × 5 mm). Therefore, the coil is actually at the edge of the Al plate in experiment setup, which leads to the decrease of the resonant frequency. Although the current verification experiment was done on a flat surface and specified dimension defects, it is feasible to detect unknown samples with varied defect dimension/wavy surface when a smaller coil is used, and an accurate automatic row and column scan system is established, which will be done in the future.

## 3. Conclusions

In summary, we have demonstrated a triboelectric nanogenerator-based self-powered resonant sensor. By integrating an inductor and microswitch with the TENG device, a very stable (~1% fluctuation) and strong (~200 V peak to peak voltage) resonant frequency of the TENG voltage output has been obtained, which is immune to the cross disturbance of contacting (force, frequency, distance) and environmental variables. It is then utilized for non-destructive defect detection in metals. The changing trend of resonant frequency varies for different types of defects, and the experiment and simulation results of non-destructive defect detection showed good consistency, demonstrating the potential of the self-powered TENG based resonant system to be used as high stability and sensitivity magnetic sensors.

## References

[1] Wang Z., Lin L., Chen J., Niu S., Zi Y. (2016). Triboelectric Nanogenerators.

[2] Nguyen V., Kelly S., Yang R. (2017). Piezoelectric peptide-based nanogenerator enhanced by single-electrode triboelectric nanogenerator. APL Mater..

[3] Han S.A., Kim T.-H., Kim S.K., Lee K.H., Park H.-J., Lee J.-H., Kim S.-W. (2018). Piezoelectric Nanogenerators: Point-Defect-Passivated MoS_2_ Nanosheet-Based High Performance Piezoelectric Nanogenerator. Adv. Mater..

[4] Lee Y.B., Han J.K., Noothongkaew S., Kim S.K., Song W., Myung S., Lee S.S., Lim J., Bu S.D., An K.-S. (2017). Toward Arbitrary-Direction Energy Harvesting through Flexible Piezoelectric Nanogenerators Using Perovskite PbTiO _3_ Nanotube Arrays. Adv. Mater..

[5] Sultana A., Alam Md.M., Middya T.R., Mandal D. (2018). A pyroelectric generator as a self-powered temperature sensor for sustainable thermal energy harvesting from waste heat and human body heat. Appl. Energy.

[6] Xue H., Yang Q., Wang D., Luo W., Wang W., Lin M., Liang D., Luo Q. (2017). A wearable pyroelectric nanogenerator and self-powered breathing sensor. Nano Energy.

[7] Wang X., Dai Y., Liu R., He X., Li S., Wang Z.L. (2017). Light-Triggered Pyroelectric Nanogenerator Based on a pn-Junction for Self-Powered Near-Infrared Photosensing. ACS Nano.

[8] Pu X., Liu M., Chen X., Sun J., Du C., Zhang Y., Zhai J., Hu W., Wang Z.L. (2017). Ultrastretchable, transparent triboelectric nanogenerator as electronic skin for biomechanical energy harvesting and tactile sensing. Sci. Adv..

[9] Lee Y., Cha S.H., Kim Y.-W., Choi D., Sun J.-Y. (2018). Transparent and attachable ionic communicators based on self-cleanable triboelectric nanogenerators. Nat. Commun..

[10] Zhu G., Lin Z.-H., Jing Q., Bai P., Pan C., Yang Y., Zhou Y., Wang Z.L. (2013). Toward Large-Scale Energy Harvesting by a Nanoparticle-Enhanced Triboelectric Nanogenerator. Nano Lett..

[11] Lin Z., Chen J., Li X., Zhou Z., Meng K., Wei W., Yang J., Wang Z.L. (2017). Triboelectric Nanogenerator Enabled Body Sensor Network for Self-Powered Human Heart-Rate Monitoring. ACS Nano.

[12] Zhang S.L., Lai Y.-C., He X., Liu R., Zi Y., Wang Z.L. (2017). Auxetic Foam-Based Contact-Mode Triboelectric Nanogenerator with Highly Sensitive Self-Powered Strain Sensing Capabilities to Monitor Human Body Movement. Adv. Funct. Mater..

[13] Bai P., Zhu G., Lin Z.-H., Jing Q., Chen J., Zhang G., Ma J., Wang Z.L. (2013). Integrated Multilayered Triboelectric Nanogenerator for Harvesting Biomechanical Energy from Human Motions. ACS Nano.

[14] Liang Q., Yan X., Liao X., Zhang Y. (2016). Integrated multi-unit transparent triboelectric nanogenerator harvesting rain power for driving electronics. Nano Energy.

[15] Zhu H.R., Tang W., Gao C.Z., Han Y., Li T., Cao X., Wang Z.L. (2015). Self-powered metal surface anti-corrosion protection using energy harvested from rain drops and wind. Nano Energy.

[16] Su Y., Wen X., Zhu G., Yang J., Chen J., Bai P., Wu Z., Jiang Y., Lin Wang Z. (2014). Hybrid triboelectric nanogenerator for harvesting water wave energy and as a self-powered distress signal emitter. Nano Energy.

[17] Seol M.-L., Woo J.-H., Jeon S.-B., Kim D., Park S.-J., Hur J., Choi Y.-K. (2015). Vertically stacked thin triboelectric nanogenerator for wind energy harvesting. Nano Energy.

[18] Huang L., Xu W., Bai G., Wong M.-C., Yang Z., Hao J. (2016). Wind energy and blue energy harvesting based on magnetic-assisted noncontact triboelectric nanogenerator. Nano Energy.

[19] Feng Y., Zhang L., Zheng Y., Wang D., Zhou F., Liu W. (2019). Leaves based triboelectric nanogenerator (TENG) and TENG tree for wind energy harvesting. Nano Energy.

[20] Zhang H., Quan L., Chen J., Xu C., Zhang C., Dong S., Lü C., Luo J. (2019). A general optimization approach for contact-separation triboelectric nanogenerator. Nano Energy.

[21] Chen J., Guo H., Ding P., Pan R., Wang W., Xuan W., Wang X., Jin H., Dong S., Luo J. (2016). Transparent triboelectric generators based on glass and polydimethylsiloxane. Nano Energy.

[22] Lu C.X., Han C.B., Gu G.Q., Chen J., Yang Z.W., Jiang T., He C., Wang Z.L. (2017). Temperature Effect on Performance of Triboelectric Nanogenerator. Adv. Eng. Mater..

[23] Wen X., Su Y., Yang Y., Zhang H., Wang Z.L. (2014). Applicability of triboelectric generator over a wide range of temperature. Nano Energy.

[24] Nguyen V., Yang R. (2013). Effect of humidity and pressure on the triboelectric nanogenerator. Nano Energy.

[25] Zheng Q., Jin Y., Liu Z., Ouyang H., Li H., Shi B., Jiang W., Zhang H., Li Z., Wang Z.L. (2016). Robust Multilayered Encapsulation for High-Performance Triboelectric Nanogenerator in Harsh Environment. ACS Appl. Mater. Interfaces.

[26] Chen J., Xuan W., Zhao P., Farooq U., Ding P., Yin W., Jin H., Wang X., Fu Y., Dong S. (2018). Triboelectric effect based instantaneous self-powered wireless sensing with self-determined identity. Nano Energy.

[27] Yin W., Xie Y., Long J., Zhao P., Chen J., Luo J., Wang X., Dong S. (2018). A self-power-transmission and non-contact-reception keyboard based on a novel resonant triboelectric nanogenerator (R-TENG). Nano Energy.

[28] Wuliang Yin, Peyton A.J., Zysko G., Denno R. (2008). Simultaneous Noncontact Measurement of Water Level and Conductivity. IEEE Trans. Instrum. Meas..

[29] García-Martín J., Gómez-Gil J., Vázquez-Sánchez E. (2011). Non-Destructive Techniques Based on Eddy Current Testing. Sensors.

[30] Mook G., Lange R., Koeser O. (2001). Non-destructive characterisation of carbon-bre-reinforced plastics by means of eddy-currents. Compos. Sci. Technol..

